# Heterodyne x-ray diffuse scattering from coherent phonons

**DOI:** 10.1063/1.4989401

**Published:** 2017-08-10

**Authors:** M. Kozina, M. Trigo, M. Chollet, J. N. Clark, J. M. Glownia, A. C. Gossard, T. Henighan, M. P. Jiang, H. Lu, A. Majumdar, D. Zhu, D. A. Reis

**Affiliations:** 1 Linac Coherent Light Source, SLAC National Accelerator Laboratory, Menlo Park, California 94025, USA; 2 Stanford PULSE Institute, SLAC National Accelerator Laboratory, Menlo Park, California 94025, USA; 3 Department of Applied Physics, Stanford University, Stanford, California 94305, USA; 4 SIMES Institute, SLAC National Accelerator Laboratory, Menlo Park, California 94025, USA; 5 Materials Department, University of California, Santa Barbara, Santa Barbara, California 93106, USA; 6 Department of Physics, Stanford University, Stanford, California 94305, USA; 7 Stanford Precourt Institute for Energy, Stanford University, Stanford, California 94305, USA; 8 Department of Mechanical Engineering and Department of Materials Science and Engineering, Stanford University, Stanford, California 94305, USA; 9 Department of Photon Science and Department of Applied Physics, Stanford University, Stanford, California 94305, USA

## Abstract

Here, we report Fourier-transform inelastic x-ray scattering measurements of photoexcited GaAs with embedded ErAs nanoparticles. We observe temporal oscillations in the x-ray scattering intensity, which we attribute to inelastic scattering from coherent acoustic phonons. Unlike in thermal equilibrium, where inelastic x-ray scattering is proportional to the phonon occupation, we show that the scattering is proportional to the phonon amplitude for coherent states. The wavevectors of the observed phonons extend beyond the excitation wavevector. The nanoparticles break the discrete translational symmetry of the lattice, enabling the generation of large wavevector coherent phonons. Elastic scattering of x-ray photons from the nanoparticles provides a reference for heterodyne mixing, yielding signals proportional to the phonon amplitude.

The temperature effect on x-ray diffraction patterns of crystals reduces the intensity of Bragg peaks by the Debye-Waller factor and produces a weak diffuse inelastic background between the peaks. The leading term of the expansion of this thermal diffuse scattering intensity I(Q) in powers of the atomic displacement *u* is $\propto 〈u^{2}〉$ because 〈u〉=0, where in this case the expectation value represents a thermal average. In the harmonic approximation, *u* is equally likely to be positive or negative, and so, ${〈u〉}^{n}=0$ for odd *n*.^1^ This constraint no longer holds for certain non-thermal states, such as coherent phonons.^2^

We recently showed for femtosecond optical excitation of single crystals that the diffuse scattering intensity I(Q,t) shows temporal oscillations related to the frequencies of phonons corresponding to momentum transfer Q.^3^ These oscillations are due to squeezed phonons with $〈U_{\lambda ,q}〉=0$ and a time-dependent $〈U_{\lambda ,q}^{2}〉$, where $U_{\lambda ,q}$ is the phonon displacement at reduced wavevector q=Q−G for the nearest reciprocal lattice vector G and branch *λ*.^4^ In contrast, a coherent phonon has time-dependent $〈U_{\lambda ,q}〉$ as well as $〈U_{\lambda ,q}^{2}〉$ although the mean-squared displacement is constant. In this case, the femtosecond x-ray diffuse scattering intensity acquires a term $\propto 〈U_{\lambda ,q}〉$ that can be comparable to the component at $〈U_{\lambda ,q}^{2}〉$.

For perfect crystals, discrete translational symmetry limits coherent phonon generation to *q* comparable to the wavevector of the light.^5^ Under optical excitation, this limits the generated phonons to be near the Brillouin zone center (q≈0). By breaking discrete translational symmetry, this restriction can be modified. For example, in the case of a sharp interface, translational symmetry is broken along the interface normal and coherent phonons can be generated with non-zero wavevectors perpendicular to the interface.^6^ These phonons have been optically generated and detected using time-resolved x-ray diffraction (see, for example, Refs. 7–11). In this work, we use nanostructured GaAs (shown in Fig. 1) to break three-dimensional discrete translational symmetry and study the phonon dynamics using time-resolved x-ray diffuse scattering.

**FIG. 1.**
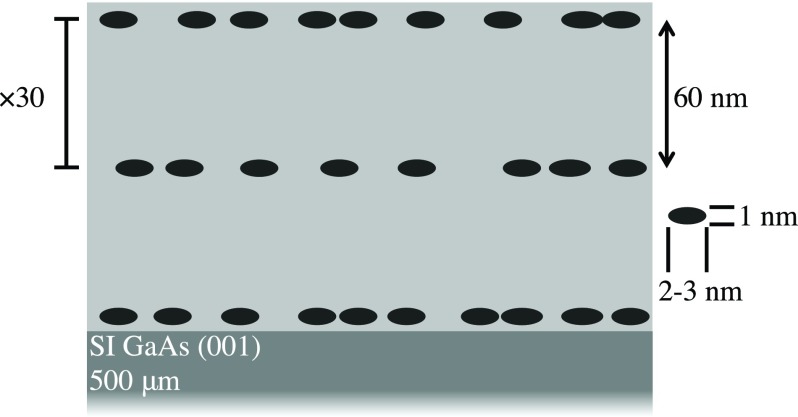
Cross-sectional diagram of the sample. The black ellipses are ErAs nanoparticles, and the gray background is GaAs. See the main text for sample details.

We report a femtosecond time-resolved measurement of the diffuse scattering from photo-excited GaAs embedded with ErAs nanoparticles^12^ using the Fourier-transform inelastic x-ray scattering (FT-IXS) technique.^3^ The presence of the nanoparticles breaks the discrete translational symmetry of the GaAs host and results in the production of high-wavevector coherent phonons. These manifest in the diffuse scattering as temporal oscillations in $〈U_{\lambda ,q}〉$, which we distinguish from the more general contribution at $〈U_{\lambda ,q}^{2}〉$ by the time-dependence. The presence of the nanoparticles further served to amplify the signal at $〈U_{\lambda ,q}〉$ through the heterodyne mixing between the inelastic scattering from the phonon and the elastic scattering from the nanoparticles.

The measurements were performed at the XPP instrument of the Linac Coherent Light Source.^13^ To excite the sample, we used ultrafast 800 nm radiation from a Ti:Sapphire laser system (120 Hz, 20 mJ, 50 fs, p-polarized). The x rays were monochromatic at 8.3 keV with an ∼0.5 eV bandwidth and 10^9^–10^10^ photons/shot.^14^ The diffuse scattering measurements were performed in a grazing-incidence geometry (∼1.3°) with the x rays and optical light nearly collinear to match penetration depths. The x-ray spot was 200 *μ*m (H) × 10 *μ*m (V) to accommodate the projection along the sample at grazing incidence. All reported measurements were performed using a 37.5 mJ/cm^2^ (incident, projected on the sample) optical fluence overfilling the x-ray spot. The sample was at room temperature and enclosed in a He environment to diminish low-angle air scattering. The scattering signal was measured using an area detector (CS-PAD^15^) on a shot-by-shot basis.

Our sample was a GaAs host with embedded ErAs nanoparticles (referred to below as the nanostructure). As a reference, we also looked at pure GaAs. Alternating layers of pure GaAs and GaAs with ErAs nanoparticles were grown epitaxially on a 500 *μ*m SI GaAs wafer [surface normal (001)]. Each repeated unit was 60 nm of GaAs with 1.2 monolayers of ErAs. There were a total of 30 units (1.8 *μ*m total thickness). We show a diagram of the sample cross-section in Fig. 1. The areal number density of the ErAs nanoparticles was $7\times 10^{12}$/cm^2^. For details of the sample growth, see Ref. 12.

Upon excitation of the nanostructure with 800 nm laser pulses, we observed temporal oscillations in the diffuse scattering intensity. In Figs. 2(A) and 2(B), we show two portions of reciprocal space captured simultaneously (that is, for a fixed experimental geometry). Each pixel of the image corresponds to a small volume of reciprocal space and can be mapped to a specific phonon wavevector q in the reduced scheme. Panel (A) corresponds to the ($\bar{1}31$) zone and panel (B) to the ($\bar{1}13$) zone (conventional unit cell). The ellipses correspond to equal contours of reduced momentum transfer in units of Å^−1^ (with the convention q=2 sin θ/λ). The black arrows *α* and *β* are paths through reciprocal space, which will be discussed below in the context of Fig. 3, while the colored symbols mark particular values of momentum transfer. The change in scattering intensity as a function of time is momentum-dependent and is illustrated in Fig. 2(C) for the four symbols in Figs. 2(A) and 2(B). The value of reduced momentum at each point in units of mÅ^−1^ is given in Fig. 2(C). Each time trace shows a few-ps rise time overlaid with rapid oscillations that persist for up to 40 ps. The rapid oscillations are from coherently excited transverse-acoustic phonons (cf. Refs. 3, 4, and 16) generated by the 800 nm light. As we describe below, these phonons have both squeezed and coherent character.

**FIG. 2.**
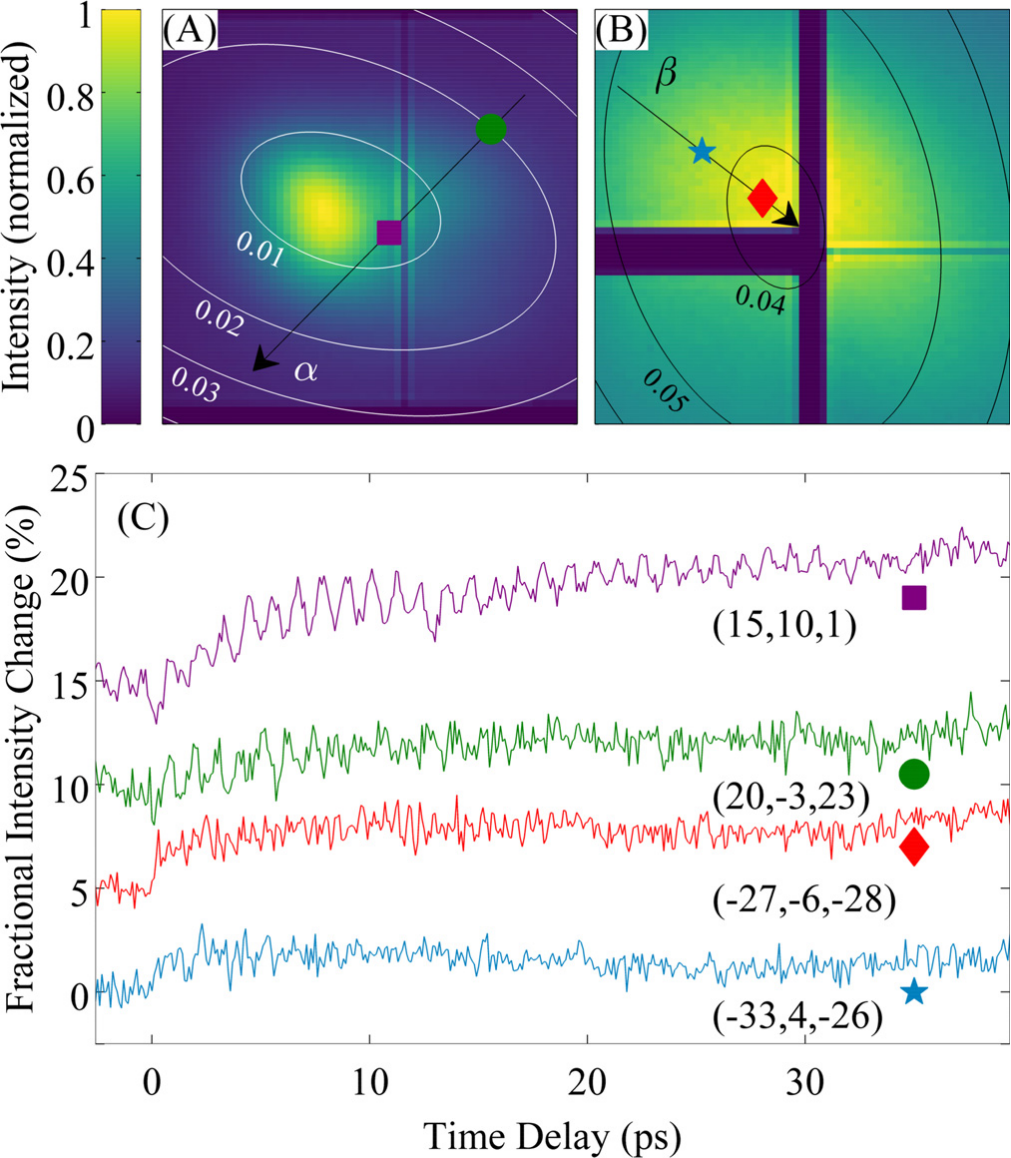
Diffuse scattering for GaAs with embedded ErAs nanoparticles from the ($\bar{1}$31) zone (A) and the ($\bar{1}$13) zone (B). The contours are values of reduced wavevector in Å^–1^. The black arrows are paths through reciprocal space considered in Fig. 3. The four shapes (purple square, green circle, red diamond, and blue star) correspond to four points in reciprocal space. The associated dynamics in the diffuse scattering intensity is shown at these four points in (C), along with the value of the reduced momentum at each point in mÅ^–1^. Traces are offset vertically for clarity.

**FIG. 3.**
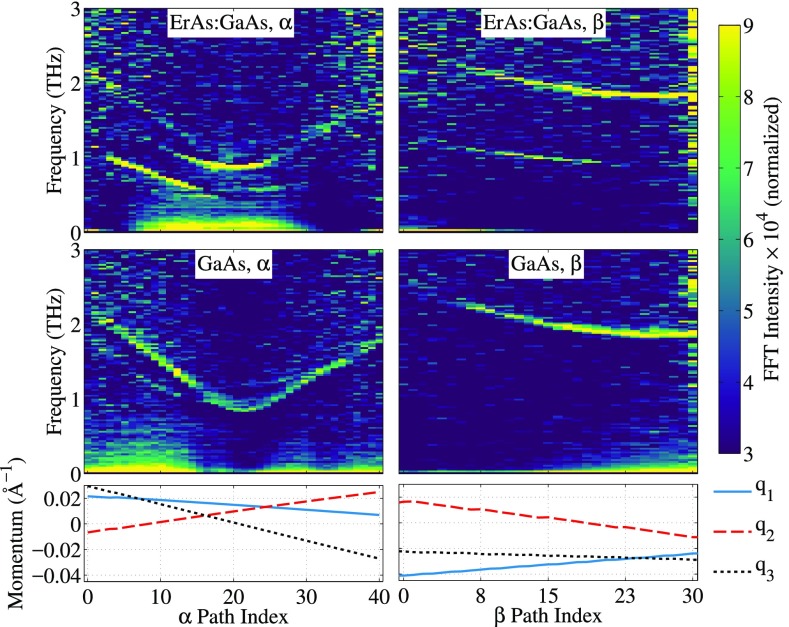
Frequency-domain analysis of time-resolved diffuse scattering data along black arrows in Figs. 2(A) and 2(B) corresponding to specific paths in reciprocal space. The color scale is the magnitude of the Fast Fourier Transform (FFT) divided by the static diffuse scattering signal (no optical excitation) and scaled by the square root of the FFT frequency (to better visualize high frequency components). Each panel is labeled by the material and the path in reciprocal space. The lines below the four panels are the coordinates of the reduced momentum transfer along each path in Figs. 2(A) and 2(B) corresponding to the points in the Brillouin zone, which are cut by a one-dimensional path in the Ewald sphere. The values on the horizontal axes index the paths in the direction of the arrows in Figs. 2(A) and 2(B).

In Fig. 3, we show the magnitude of the Fast Fourier Transform (FFT) of the time-resolved change in scattering intensity for two paths through the Brillouin zone for both the nanostructure (top panels, labeled ErAs:GaAs) and the GaAs reference (bottom panels, labeled GaAs). The magnitude of the FFT is divided by the static diffuse scattering signal (no optical excitation) and scaled by the square root of the FFT frequency (to better visualize high frequency components). We show data along the black arrows in Figs. 2(A) and 2(B). The first path (*α*) passes close to the Brillouin zone center, while the second (*β*) is further away. The data in the left column are along *α*, while the data in the right column are along *β*. Note that the two paths (*α*, *β*) represent arcs in reciprocal space, and thus, three coordinates are needed to specify q. We parameterize these paths with an index indicated on the bottom of Fig. 3. The direction of the arrow in Fig. 2 indicates the direction of the increasing index. Along *α*, we see a clear dispersive feature in both the nanostructure and the pure GaAs samples ranging from 1 THz to 2 THz which we attribute to coherently excited modes in the transverse acoustic branch that modulate $〈U_{\lambda ,q}^{2}〉$ at twice the phonon frequency.^3,4^ There is also a noticeable break in the dispersive feature near the path index of 15 at approximately 1 THz, which indicates the splitting between the two non-degenerate transverse acoustic branches. Similarly, along *β*, we observe intensity ranging from 1.8 to 2.5 THz. This frequency range is higher than that along *α* because the *β* path is further from the Brillouin zone center.

However, there are additional dispersive features in the nanostructure scattering data that are not present in pure GaAs, which we attribute to large-wavevector coherent phonons. Specifically, the data for the nanostructure along *α* show a dispersive feature from path indices 3–15 corresponding to the phonon frequency. Along *β* from indices 5–22, we similarly observe signals at the phonon frequency in the nanostructure. These bands are markedly absent in the GaAs reference sample. We attribute these lower-frequency oscillations to the presence of large-wavevector coherent phonons, enabled by breaking three-dimensional discrete translational symmetry with the nanoparticles.

In order to understand the detection of the additional dispersive bands, we consider the scattered x-ray electric field from the nanostructure. Both GaAs and ErAs form on the FCC lattice. For the zinc blende GaAs, in fractional coordinates, As is at the (0,0,0) site and Ga is at $\left(\frac{1}{4},\frac{1}{4},\frac{1}{4}\right)$. In ErAs, the structure is rock-salt type and As occupies the same (0,0,0) location, while Er is at $\left(\frac{1}{2},\frac{1}{2},\frac{1}{2}\right)$. The epitaxial growth process ensures that As in the ErAs nanoparticles lies on the same sublattice as As in the GaAs host.^17,18^ Thus to calculate the total x-ray scattering, we account for the presence of the nanoparticles in the GaAs host by replacing clusters of Ga ions with Er, with the appropriate real-space displacement to account for different bases. The scattered field at momentum transfer Q,

$$\begin{array}{c} E(Q)\propto \sum_{j,s}f_{s}e^{2\pi iQ\cdot (R_{j}+\delta_{s}+u_{j,s})}+\sum_{j\prime }f_{D}e^{2\pi iQ\cdot (R_{j^{\prime }}+\delta_{\text{Ga}}+u_{j^{\prime },\text{Ga}})}, \end{array}$$ (1)

where *f_s_*, $R_{j},\;\delta_{s}$, and $u_{j,s}$ are the atomic scattering factor (with an implicit Q-dependence), Bravais lattice vector, basis vector, and site-dependent deviation from equilibrium, respectively, for ion *s* in unit cell *j* (cf. Ref. 19). The first sum is over all cells *j* in a perfect GaAs lattice with s= Ga and As. The second sum is only over unit cells j′, where Er has replaced Ga to form nanoparticles. Because the nanoparticles replace Ga with Er, we avoid over-counting the removed Ga from the host by defining an effective scattering factor for Er,

$$f_{D}=f_{\text{Er}}e^{2\pi iQ\cdot (\delta_{\text{Er}}-\delta_{\text{Ga}})}-f_{\text{Ga}}.$$ (2)

By assuming that the deviation $u_{j,s}$ from perfect crystallinity is small, we can approximate each summand to linear order in $u_{j,s}$. Moreover, we exploit the periodicity of the As sublattice to expand

$$u_{j,s}=\frac{1}{\sqrt{N}}\sum_{\lambda ,q}U_{\lambda ,q}e^{2\pi iq\cdot (R_{j}+\delta_{s})},$$ (3)

where *N* is the total number of As sites. $U_{\lambda ,q}$ corresponds to the phonon amplitude at reduced wavevector q and branch *λ* and has the implicit time-dependence $\text{exp}\;(-i\omega_{\lambda ,q}t)$, where $\omega_{\lambda ,q}$ is the phonon frequency. Because the perturbation $u_{j,s}$ is real, $U_{\lambda ,-q}=U_{\lambda ,q}^{*}$. We then substitute this expression into Eq. (2) and find (see supplementary material for detailed calculations)

$$\begin{array}{c} E(Q)\propto F(Q)S(Q)+\frac{2\pi i}{\sqrt{N}}\sum_{\lambda ,q}\left(Q\cdot U_{\lambda ,q})F(Q+q)S(Q+q\right)\\ +F_{N}(Q)S_{N}(Q)+\frac{2\pi i}{\sqrt{N}}\sum_{\lambda ,q}\left(Q\cdot U_{\lambda ,q})F_{N}(Q+q)S_{N}(Q+q\right). \end{array}$$ (4)

Here, F(Q) and $F_{N}(Q)$ correspond to the structure factor for a single GaAs unit cell and an average ErAs nanoparticle treated as a single large unit cell, respectively, at momentum transfer Q [see Eq. (6) in the supplementary material]. We approximate all nanoparticles to be identical in size (1 nm cross-plane, 2–3 nm in-plane) and orientation (because of the epitaxial growth) but located at random sites throughout the GaAs host.^20–23^ The function S(Q) is the lattice sum, while $S_{N}(Q)$ is a truncated version of the lattice sum over only the nanoparticle positions [see Eq. (6) in the supplementary material]. Because the nanoparticles are small, $F_{N}(Q)$ varies slowly throughout the Brillouin zone. Similarly, $S_{N}(Q)$ varies more slowly about the Bragg condition Q=G than S(Q). The first and third terms of Eq. (4) correspond to elastic scattering at momentum transfer Q, while the second and fourth terms account for inelastically scattered components at Q+q.

To calculate the scattered intensity, it is necessary to multiply Eq. (4) by its complex conjugate, resulting in several cross-terms. Here, we consider only terms linear in $U_{\lambda ,q}$. These correspond to a heterodyne interference between an elastic component at Q and an inelastic component at Q+q, resulting in scattered photons that are offset in frequency by $\omega_{\lambda ,q}$. Guided by one-dimensional simulations (see supplementary material Fig. 1), we expect that the interference term at a particular Q+q that has the largest contribution is that which mixes the elastic contribution from the nanoparticle at Q with the inelastic contribution from the host at Q+q, namely, $ℜe[S^{*}(Q+q)S_{N}(Q)]$. This corresponds to heterodyne detection where the elastic scattering from the nanoparticle acts as the local oscillator. We expect this particular cross-term to be the largest because the truncated lattice sum $S_{N}(Q)$ for the nanoparticle is much broader in Q than the full lattice sum S(Q). Experimentally, we directly compare the nanoparticle and host scattering terms by exploiting the difference between the zinc blende (GaAs) and rock salt (ErAs) structures.

In Fig. 4, we show the static diffuse scattering from the nanostructure primarily in the ($\bar{2}$22) zone (conventional unit cell) very close to the zone center. Panel (A) shows the full projection of the zone captured by the detector, while (B) shows a zoomed-in region. For rock salt, the ($\bar{2}$22) reflection is strong, while it is weak for zinc blende. Thus, despite the larger volume of GaAs illuminated by the x rays, the scattering signal from ErAs is much more apparent than that in a zone for which both structures have strong contributions [cf. the $\left(\bar{1}31\right)$ zone in Fig. 2(A)]. As seen in Fig. 4(A), there is non-negligible scattering across a large fraction of the Brillouin zone extending out to 0.025 Å^–1^, primarily from the ErAs nanoparticles and directly illustrating the slow variation of $F_{N}(Q)S_{N}(Q)$. On the other hand, the function S(Q+q)∼δ(G−Q−q) because of the large number of GaAs unit cells, thus selecting phonons with a well-defined wavevector q. The ErAs scattering therefore acts as a static background over a large region of the Brillouin zone, which can mix with the time-varying GaAs diffuse scattering, yielding a heterodyne contribution to the signal. Furthermore, we confirm our approximations of a narrow size distribution and orientational arrangement of the nanoparticles by resolving fringes with spatially varying lobes in the periphery of the scattering [Fig. 4(B)].

**FIG. 4.**
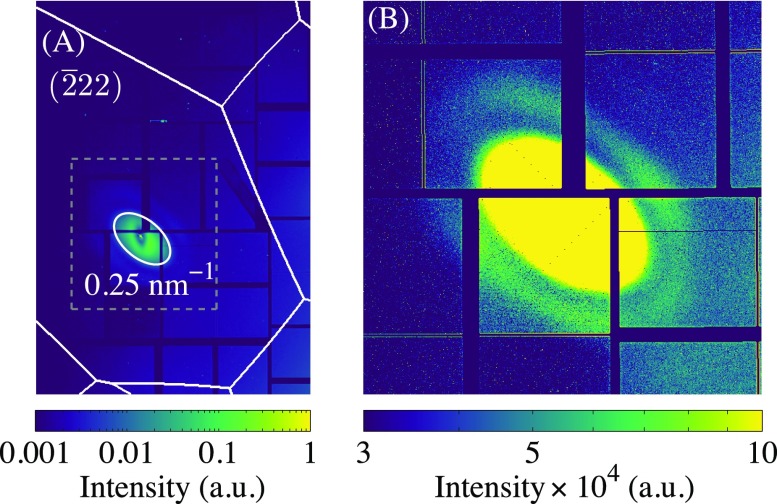
(A) Static diffuse scattering image of the ($\bar{2}22)$ Brillouin zone for GaAs with embedded ErAs nanoparticles. The white ring corresponds to a contour at 0.025 Å^–1^ of the reciprocal lattice vector magnitude (reduced zone). The white lines denote the Brillouin zone boundaries. The gray dashed box shows the zoom region. (B) The same data as in (A) zoomed in to the center of the ($\bar{2}22)$ Brillouin zone [gray dashed box in (A)] and with a color scale saturated to emphasize the fine features in the periphery.

In order to observe the heterodyne signal, it is necessary to induce perturbations that enable $〈U_{\lambda ,q}〉(t)\neq 0$. This is achieved, for example, via the excitation of a coherent phonon state.^2^ Coherent phonon modes cannot be induced at large wavevectors in perfectly periodic crystals by optical light because of crystal momentum conservation.^5^ The ErAs nanoparticles however break the three-dimensional discrete translational symmetry of the system^24^ and therefore allow the generation of coherent phonons with wavevectors in all directions. We thus attribute the temporal oscillations at the phonon frequency to coherent phonons observed through the heterodyne detection mechanism. In pure GaAs, however, we only observe temporal oscillations at twice the phonon frequency. Because of long range order, optical radiation cannot generate large wavevector coherent phonons except along the surface normal due to the sharp air/sample interface. However, our measurements do not probe wavevectors along this direction. Thus, we attribute the signal in the pure GaAs to squeezed phonons consistent with previous reports.^3,4^

We have shown that coherent phonons with large wavevectors are generated by ultrafast optical excitation in GaAs with embedded ErAs nanoparticles. The nanoparticles break the three-dimensional discrete translational symmetry of the GaAs host and so enable coherent phonon generation at large wavevectors in all directions. Moreover, x-ray elastic scattering from the nanostructure interferes with the x-ray inelastic diffuse scattering to provide a heterodyne signal at large wavevectors that oscillates at the phonon frequency and is proportional to the amplitude of the phonon mode. Our results show that a small amount of disorder is not detrimental to FT-IXS but in fact can be beneficial due to the signal enhancement from the heterodyne contribution. This represents a first step towards extending FT-IXS for studying vibrational dynamics of disordered materials including alloys and glasses.

See supplementary material for the detailed derivation of Eq. (4) and for the one-dimensional simulation of the nanoparticle scattering.
